# T‐cell activation by transgenic rice seeds expressing the genetically modified Japanese cedar pollen allergens

**DOI:** 10.1111/imm.13097

**Published:** 2019-08-23

**Authors:** Shinya Takaishi, Saburo Saito, Tomonori Endo, Daiya Asaka, Yuhya Wakasa, Hidenori Takagi, Kenjiro Ozawa, Fumio Takaiwa, Nobuyoshi Otori, Hiromi Kojima

**Affiliations:** ^1^ Department of Otorhinolaryngology Jikei University School of Medicine Tokyo Japan; ^2^ Division of Molecular Immunology Research Center for Medical Sciences Jikei University School of Medicine Tokyo Japan; ^3^ Plant Molecular Farming Unit Division of Biotechnology Institute of Agrobiological Sciences National Agriculture and Food Research Organization Ibaraki Japan

**Keywords:** immunotherapy, pollinosis, T‐cell epitope, T‐cell line, transgenic rice

## Abstract

Transgenic rice seeds that contain genetically modified Cry j 1 and Cry j 2, the two major allergens of *Cryptomeria japonica* (Japanese cedar; JC), have been developed as immunotherapeutic candidates for JC pollinosis. Because the transgenic rice (TG‐rice) seeds express allergens containing whole amino acid sequences of Cry j 1 and Cry j 2 in the endosperm tissue (edible part of rice grain), they can potentially target all Cry j 1‐ and Cry j 2‐specific T‐cells. However, it was unknown whether antigenicity of Cry j 1 and Cry j 2 could be completely preserved in TG‐rice seeds. We verified the antigenicity of TG‐rice seeds to T‐cells through the analysis of the proliferative responses of T‐cells in Cry j 1‐ or Cry j 2‐immunized mice or T‐cell lines to TG‐rice seed extract. First, four mouse strains were immunized with Cry j 1 or Cry j 2. T‐cells in the immunized mice proliferated on treatment with TG‐rice seed extract, but not non‐transgenic wild‐type rice (WT‐rice) seed extract. Furthermore, T‐cell lines were established from the spleen cells of the immunized mice. Each T‐cell line resulted in a proliferative response to TG‐rice seed extract, but not to WT‐rice seed extract, suggesting that TG‐rice seeds certainly express T‐cell epitopes corresponding to T‐cell lines. Considering the modified amino acid sequences of Cry j 1 and Cry j 2 in TG‐rice seeds, the expression of specific T‐cell epitopes suggested that TG‐rice seeds express all possible T‐cell epitope repertoires of Cry j 1 and Cry j 2.

**Abbreviations**
APCs: antigen‐presenting cells
IgE: immunoglobulin E
JC: Japanese cedar
PBS : phosphate‐buffered saline
RPMI : Roswell Park Memorial Institute
S.I.: stimulation index
SCIT: subcutaneous immunotherapy
SLIT: sublingual immunotherapy
TG‐rice : transgenic rice
WT‐rice: non‐transgenic wild‐type rice

## Introduction

Japanese cedar (JC; *Cryptomeria japonica*) pollinosis is one of the most serious type I allergic diseases in Japan, which causes allergic rhinitis, conjunctivitis and asthma as clinical symptoms in the period of February to April each year. An epidemiological study conducted in 2008 indicated that the prevalence of JC pollinosis was 26·5%.^1^ Conventional therapies for JC pollinosis include allergen avoidance, pharmacotherapy using antihistamines or corticosteroid nasal spray, and specific immunotherapy.^2^ Allergen‐specific immunotherapy is based on the administration of disease‐eliciting allergens to induce a state of unresponsiveness toward them. Subcutaneous immunotherapy (SCIT) has been used for a long time as a desensitizing therapy for JC pollinosis. Sublingual immunotherapy (SLIT) was introduced to the treatment of JC pollinosis in 2014.^3^

Recently, transgenic rice seeds that contain genetically modified Cry j 1 and Cry j 2, the two major allergens of *C. japonica* (JC), have been developed as immunotherapeutic candidates for JC pollinosis. More than 90% of patients suffering from JC pollinosis have immunoglobulin E (IgE) specific to both Cry j 1 and Cry j 2, and the remainder carry an IgE specific to only one of these two allergens.^4^ The transgenic rice (TG‐rice) seeds express allergens containing whole amino acid sequences of Cry j 1 and Cry j 2 in the endosperm tissue (edible part of rice grain): Cry j 1 gene was divided into three overlapping fragments, and the amino acid sequence of Cry j 2 gene was shuffled.^5^, ^6^, ^7^ Wakasa *et al*.^5^ demonstrated the accumulation of Cry j 1 and Cry j 2 in TG‐rice seeds by immunoblot analysis using anti‐glutelin‐specific (GluA, GluB and GluC) and anti‐Cry j 2 antibodies after electrophoresis of the seed proteins on sodium dodecyl sulphate–polyacrylamide gel electrophoresis (SDS‐PAGE). Intracellular localization of modified Cry j 1 and Cry j 2 in endosperm cells was also observed by immunoelectron microscopy.^5^ The allergens in TG‐rice seeds were engineered to engage T‐cell receptors, but they were of insufficient length to cross‐link IgE on the surface of mast cells or basophils. However, the actual IgE‐binding and IgE‐crosslinking abilities of the antigens in TG‐rice seeds remain unverified. We previously revealed that the modified allergens in TG‐rice seeds had a low risk of IgE‐mediated adverse events by evaluating basophil activation of patients with JC pollinosis after *in vitro* stimulation with TG‐rice seed extract in a basophil activation test.^8^

Because TG‐rice seeds contain whole amino acid sequences of Cry j 1 and Cry j 2, it is possible that all types of Cry j 1‐ or Cry j 2‐specific T‐cells could be targeted. Although the efficacy of oral immunotherapy with TG‐rice seeds has already been demonstrated in mouse models,^9^, ^10^, ^11^ it is unknown whether Cry j 1 and Cry j 2 antigenicity is completely preserved in TG‐rice seeds. Accordingly, the aim of this study was to prove the antigenicity of TG‐rice seeds to Cry j 1‐ or Cry j 2‐specific T‐cells by analysing the proliferative responses of T‐cells in Cry j 1‐ or Cry j 2‐immunized mice or established T‐cell lines to TG‐rice seed extract.

## Materials and methods

Four mouse strains were immunized with Cry j 1 or Cry j 2, and their T‐cell proliferation assays were conducted to assess the antigenicity of TG‐rice seed extract. T‐cell epitope sites in Cry j 1‐ or Cry j 2‐immunized mice were identified using overlapping peptides spanning the entire sequences of Cry j 1 or Cry j 2. Next, we established five types of T‐cell lines, based on the spleen cells of Cry j 1‐ or Cry j 2‐immunized mice. T‐cell line proliferation assays were conducted to prove the expression of specific T‐cell epitopes in TG‐rice seeds. Furthermore, the proliferative responses of T‐cell lines to boiled‐TG‐rice seed extract were examined to verify whether TG‐rice seeds retain antigenicity to T‐cells after boiling. This study was approved by the Institutional Animal Care and Use Committee of the Jikei University [identification (ID): 2016‐091]. The care and handling of the mice followed the Animal Experimentation Guidelines of Jikei University School of Medicine.

#### Allergen extraction from the protein body powder of TG‐rice seeds

Transgenic‐rice seeds deposit the recombinant Cry j 1 and Cry j 2 in ER‐derived protein bodies in the endosperm. The protein bodies were isolated from TG‐rice seeds (Ozeki, Nishinomiya, Japan) and modified to make them powdery. Soluble allergens were extracted from powdered protein bodies as follows. First, the powdered protein bodies were dissolved in phosphate‐buffered saline (PBS) at a 1 : 150 ratio (w/v), and the mixture was sonicated on ice. Thereafter, the mixture was centrifuged at 5800 *g* for 10 min at 4°, and the supernatant was collected. The supernatant was then dialysed in PBS, concentrated 10‐fold using an Amicon Ultra‐15 Centrifugal Filter Unit (Merck Millipore, Co. Cork, UK), and sterilized through a 0·22‐μm Sterile Millex Filter Unit (Merck Millipore, Co.) to produce a filtered‐ and concentrated‐TG‐rice seed extract. Extraction from the protein body powder of non‐transgenic wild‐type rice (WT‐rice) seeds was performed in the same manner as extraction from the protein body powder of TG‐rice seeds.

#### Immunization of mice

Male BALB/c, B10.S, C57BL/6 and C3H/He mice were purchased from Sankyo Labo Service (Tokyo, Japan) and housed in our facilities under specific pathogen‐free conditions. All mice were used for experiments at the age of 6–10 weeks. The four strains of mice were injected with 10 μg of Cry j 1 or Cry j 2 antigen (Hayashibara Biochemical Laboratories, Okayama, Japan) along with 1 mg of Al(OH)_3_ as adjuvant by intraperitoneal injection twice on days 0 and 14 (*n* = 3 per each strain). On day 28, spleen cells were collected from the mice for analysis.

#### T‐cell proliferative response to TG‐rice seed extract

Extracts from WT‐ or TG‐rice seeds were added to the spleen cells of Cry j 1‐ or Cry j 2‐immunized mice. The proliferative responses of the spleen cells were determined by an *in vitro* radioactively labelled thymidine incorporation assay. Roswell Park Memorial Institute (RPMI)‐1640 medium (Gibco, Thermo Fisher Scientific, Waltham, MA) supplemented with 1% normal mouse serum was used to suspend the spleen cells. Briefly, 8 × 10^5^ spleen cells were seeded into each well of a Nunc Microwell 96‐Well Microplate (Thermo Fisher Scientific) and cultured in a 1 : 40 dilution of WT‐ or TG‐rice seed extract and several concentrations of Cry j 1 or Cry j 2 antigen in 5% CO_2_ for 72 hr at 37°. Each well was then pulsed with 0·5 μCi of ^3^H‐thymidine (American Radiolabeled Chemicals, Saint Louis, MO), and the cells were harvested 16 hr later with a Skatron Micro96 Cell Harvester (Skatron, Newmarket, UK). The level of ^3^H‐thymidine incorporation was determined by measuring the radioactivity with an LSC‐6000 liquid scintillation counter (ALOKA, Tokyo, Japan). The results were expressed as the stimulation index (S.I.), which was calculated as follows: mean counts per minute in the presence of the antigen/mean counts per minute in the absence of the antigen.

#### Identification of T‐cell epitope sites in the Cry j 1‐ or Cry j 2‐immunized mice

The T‐cell epitope sites in the immunized mice were identified using overlapping peptides spanning the entire sequences of Cry j 1 or Cry j 2. The Cry j 1 and Cry j 2 amino acid sequences were divided into 41 and 46 overlapping peptides, respectively, each composed of almost 32 residues and shifting every eight residues (Selleck Chemicals, Houston, TX). The spleen cells of the immunized mice (*n* = 3 per strain) were cultured with the overlapping peptides, and T‐cell proliferative responses to the overlapping peptides were determined by a ^3^H‐thymidine incorporation assay as described above.

#### Establishment of T‐cell lines

Cry j 1‐specific T‐cell lines were established from the spleen cells of Cry j 1‐ immunized B10.S or BALB/c mice, and Cry j 2‐specific T‐cell lines were established from the spleen cells of Cry j 2‐immunized BALB/c or C57BL/6 mice. The spleen cells were collected from the immunized mice on day 28 and suspended at a density of 4 × 10^6^/ml in RPMI‐1640 medium (Gibco, Thermo Fisher Scientific) supplemented with 1% normal mouse serum. Then, 8 × 10^6^ spleen cells were seeded into each well of a 24‐well Nunc Cell‐Culture Treated Multidish (Thermo Fisher Scientific.) and cultured with 2·5 μg/ml of Cry j 1 or Cry j 2 antigen in 5% CO_2_ at 37°. After 7 days, the cells were harvested and then cultured with each antigen in the presence of 40 μg/ml of mitomycin C (Kyowa Hakko Kogyo, Tokyo, Japan)‐treated spleen cells of syngeneic mice as antigen‐presenting cells (APCs). After more than seven cycles of antigen stimulation once every 2 or 3 weeks in the presence of the APCs, five types of T‐cell lines were established, and their antigen specificity was verified using the overlapping peptides spanning the entire sequences of Cry j 1 or Cry j 2. In the case of the T‐cell line established from Cry j 2‐immunized C57BL/6 mice, two dominant T‐cell epitope sites were observed after Cry j 2‐stimulation. Subsequently, two types of T‐cell lines were established after separate repetition of stimulation with p233‐264 or p353‐384 epitope peptides of the Cry j 2 amino acid sequences. The proliferative responses of the T‐cell lines to the overlapping peptides were determined by an *in vitro* ^3^H‐thymidine incorporation assay. Briefly, the overlapping peptides were added to each T‐cell line (1 × 10^4^ cells) in the presence of spleen cells of syngeneic mice (6 × 10^5^ cells) as APCs in 96‐well microplates and cultured in 5% CO_2_ for 48 hr at 37°. Each well was then pulsed with 0·5 μCi of ^3^H‐thymidine, and the cells were harvested 16 hr later with a Skatron Micro96 Cell Harvester. The level of ^3^H‐thymidine incorporation was determined by measuring the radioactivity with an LSC‐6000 liquid scintillation counter.

#### T‐cell line proliferative response to TG‐rice seed extract

T‐cell line proliferation was assessed to prove the expression of specific T‐cell epitopes in TG‐rice seeds via a ^3^H‐thymidine incorporation assay. The 1 : 40 dilution of WT‐ or TG‐rice seed extract was added to each T‐cell line (1 × 10^4^ cells) in the presence of spleen cells of syngeneic mice (6 × 10^5^ cells) as APCs in 96‐well microplates and cultured in 5% CO_2_ for 48 hr at 37°. Each well was then pulsed with 0·5 μCi of ^3^H‐thymidine. The cells were harvested 16 hr later, and the level of ^3^H‐thymidine incorporation was measured.

#### Evaluation of antigenicity of TG‐rice seeds after boiling

Established Cry j 1‐ and Cry j 2‐specific T‐cell lines were used to evaluate the antigenicity of TG‐rice seeds after boiling. Briefly, the 1 : 40 dilution of WT‐ or TG‐rice seed extract was boiled at 100° for 30 min and added to each T‐cell line (1 × 10^4^ cells) in the presence of spleen cells of syngeneic mice (6 × 10^5^ cells) as APCs in 96‐well microplates and cultured in 5% CO_2_ for 48 hr at 37°. Each well was then pulsed with 0·5 μCi of ^3^H‐thymidine. The cells were harvested 16 hr later, and the level of ^3^H‐thymidine incorporation was measured.

## Results

### T‐cells in the Cry j 1‐ or Cry j 2‐immunized mice showed proliferative responses to TG‐rice seed extract

**Figure 1:**
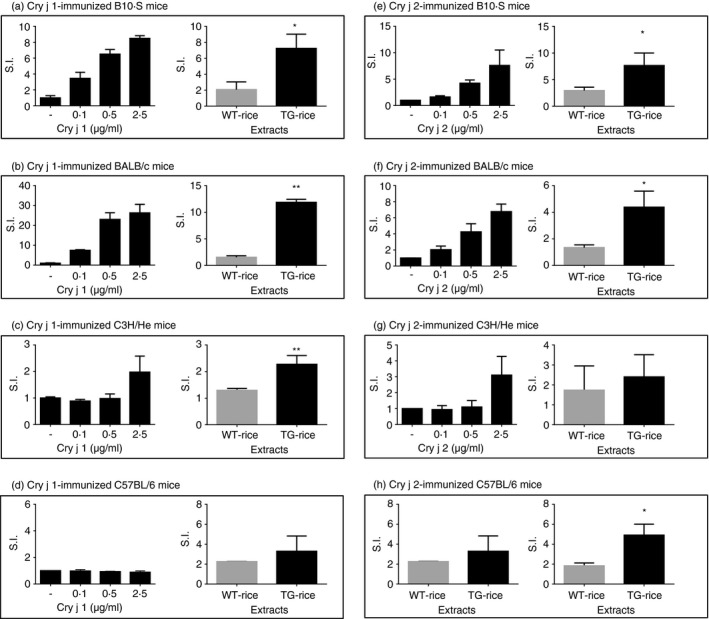
The proliferative responses of the spleen cells of Cry j 1‐ or Cry j 2‐immunized mice to non‐transgenic wild‐type rice (WT‐) or transgenic rice (TG‐rice) seed extract. Fourteen days after the last immunization with Cry j 1 or Cry j 2, the spleen cells of B10.S, BALB/c, C3H/He and C57BL/6 mice (*n* = 3 per strain) were cultured with WT‐ or TG‐rice seed extract and several concentrations of Cry j 1 or Cry j 2 antigen for 72 hr (a–h), and then pulsed with 0·5 μCi of ^3^H‐thymidine. The results of T‐cell proliferation were expressed as the stimulation index (S.I.), which was calculated as follows: mean counts per minute in the presence of the antigen divided by mean counts per minute in the absence of the antigen. *P < 0·05; *^*^ *P* < 0·01.

First, to investigate whether Cry j 1‐ or Cry j 2‐specific T‐cells were induced in Cry j 1‐ or Cry j 2‐immunized mice, the proliferative responses of the spleen cells to Cry j 1 or Cry j 2 antigen were assessed. The spleen cells of Cry j 1‐immunized B10.S, BALB/c and C3H/He mice responded to Cry j 1 antigen in a dose‐dependent manner (Fig. 1a–c). However, those of C57BL/6 mice immunized with Cry j 1 did not respond to Cry j 1 antigen, even at high concentrations (Fig. 1d), suggesting that Cry j 1‐specific T‐cells were induced in B10.S, BALB/c and C3H/He mice; C57BL/6 mice were non‐responders to Cry j 1. In contrast, the spleen cells of Cry j 2‐immunized B10.S, BALB/c, C3H/He and C57BL/6 mice responded to Cry j 2 antigen in a dose‐dependent manner (Fig. 1e–h), suggesting that Cry j 2‐specific T‐cells were induced in these four mouse strains. Thereafter, to verify the antigenicity of TG‐rice seeds, the proliferative responses of the spleen cells to WT‐ or TG‐rice seed extract were assessed. Cry j 1‐specific T‐cells derived from Cry j 1‐immunized B10.S, BALB/c and C3H/He mice showed significant proliferative responses to TG‐rice seed extract compared with those to WT‐rice seed extract (Fig. 1a–c). Similarly, Cry j 2‐specific T‐cells derived from Cry j 2‐immunized B10.S, BALB/c and C57BL/6 mice showed significant proliferative responses to TG‐rice seed extract compared with those to WT‐rice seed extract (Fig. 1e,f,h). Therefore, evident Cry j 1‐ or Cry j 2‐specific T‐cell proliferative responses to TG‐rice seed extract rather than WT‐rice seed extract were demonstrated, except in Cry j 1‐immunized C57BL/6 mice (non‐responders to Cry j 1) and Cry j 2‐immunized C3H/He mice. These results indicated that the TG‐rice seeds were sufficiently antigenic to activate Cry j 1‐ or Cry j 2‐specific T‐cells.

### T‐cell epitope sites in the Cry j 1‐ or ‐Cry j 2‐immunized mice

**Figure 2:**
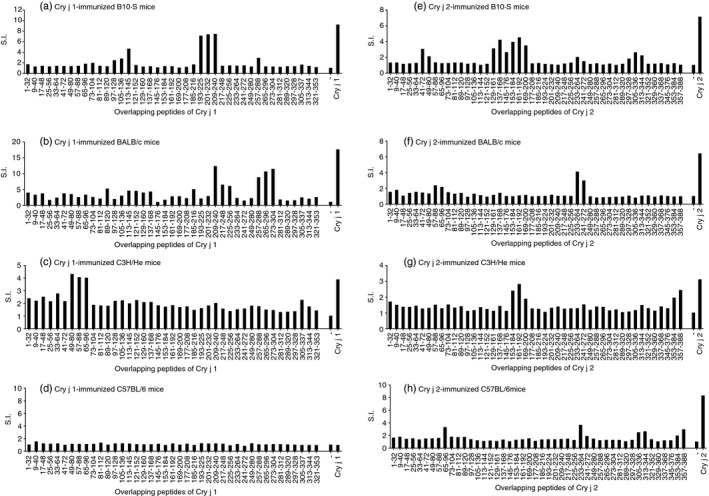
T‐cell epitopes in Cry j 1‐ or Cry j 2‐immunized mice. The overlapping peptides spanning the Cry j 1 or Cry j 2 sequences were designated by the numbers of N‐ and C‐terminal amino acid residues. The spleen cells of the Cry j 1‐immunized mice were cultured with the 41 overlapping peptides of Cry j 1 (a–d). The spleen cells of the Cry j 2‐immunized mice were cultured with the 46 overlapping peptides of Cry j 2 (e–h). T‐cell proliferative responses were determined by ^3^H‐thymidine incorporation assay as described in the legend of Fig. 1.

The proliferative responses of the spleen cells to the overlapping peptides spanning the Cry j 1 or Cry j 2 sequences were examined to determine T‐cell epitope sites in the immunized mice. T‐cells induced in the Cry j 1‐ or Cry j 2‐immunized mice reacted with various overlapping peptides of Cry j 1 or Cry j 2. The number of major T‐cell epitope sites was two in the Cry j 1‐immunized B10.S and BALB/c mice (Fig. 2a,b), and one in the Cry j 1‐immunized C3H/He mice (Fig. 2c). No T‐cell epitope site was observed in the Cry j 1‐immunized C57BL/6 mice because they were non‐responders to Cry j 1 (Fig. 2d). The number of major T‐cell epitope sites was four in the Cry j 2‐immunized B10.S (Fig. 2e) and C57BL/6 mice (Fig. 2h), and two in the Cry j 2‐immunized BALB/c (Fig. 2f) and C3H/He (Fig. 2g) mice. Considering T‐cell proliferative responses of Cry j 1‐ or Cry j 2‐immunized mice to TG‐rice seed extract (Fig. 1), these results revealed the possibility that TG‐rice seeds contain multiple epitopes of Cry j 1 and Cry j 2.

### T‐cell epitopes of Cry j 1‐ or Cry j 2‐specific T‐cell lines

**Figure 3:**
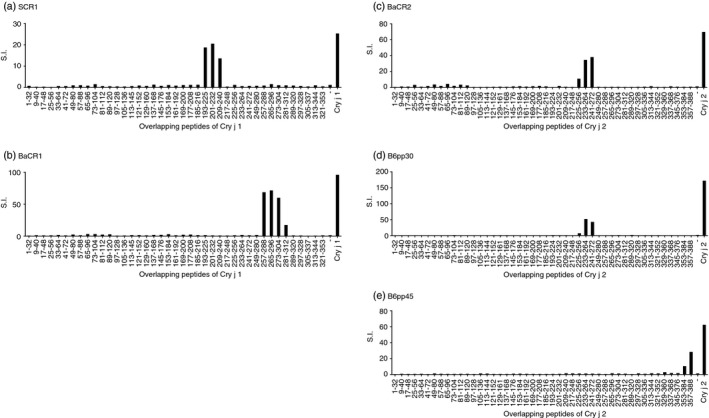
T‐cell epitopes of Cry j 1‐ or Cry j 2‐specific T‐cell lines. Cry j 1‐specific T‐cell lines were established from the spleen cells of Cry j 1‐immunized B10.S or BALB/c mice, and Cry j 2‐specific T‐cell lines were established from the spleen cells of Cry j 2‐immunized BALB/c or C57BL/6 mice, as described in the Materials and methods. The established T‐cell lines were designated as SCR1, BaCR1, BaCR2, B6pp30 and B6pp45, respectively (a–e). The proliferative responses of T‐cell lines to the overlapping peptides spanning the Cry j 1 or Cry j 2 sequences were determined by ^3^H‐thymidine incorporation assay as follows: the overlapping peptides were added to each T‐cell line in the presence of spleen cells of syngeneic mice as antigen‐presenting cells (APCs) in 96‐well microplates and cultured for 48 hr. Each well was then pulsed with 0·5 μCi of ^3^H‐thymidine and the cells were harvested 16 hr later.

To identify the T‐cell epitope of each T‐cell line, we investigated their proliferative responses to the overlapping peptides spanning the entire sequences of Cry j 1 or Cry j 2. The established T‐cell lines were designated as SCR1, BaCR1, BaCR2, B6pp30 and B6pp45. SCR1 was a T‐cell line established from the Cry j 1‐immunized B10.S mice. The proliferative responses of SCR1 to the Cry j 1‐overlapping peptides were examined. SCR1 specifically responded to p193–225, p201–232 and p209–240 Cry j 1‐overlapping peptides (Fig. 3a). Therefore, SCR1 was thought to respond to the common core epitope contained in p209–225 (DDKSMKVTVAFNQFGPN) (Table 1). BaCR1, a T‐cell line established from the Cry j 1‐immunized BALB/c mice, specifically responded to p257–288, p265–296 and p273–304 Cry j 1‐overlapping peptides (Fig. 3b) and, therefore, was thought to respond to the common core epitope contained in p273–288 (ESYKKQVTIRIGCKTS) (Table 1). Similarly, BaCR2, a T‐cell line established from the Cry j 2‐immunized BALB/c mice, specifically responded to p225–256, p233–264 and p241–272 Cry j 2‐overlapping peptides (Fig. 3c) and, therefore, was thought to respond to the common core epitope contained in p241–256 (GRENSRAEVSYVHVNG) (Table 1). B6pp30 and B6pp45 were two kinds of T‐cell lines established from the Cry j 2‐immunized C57BL/6 mice. B6pp30 specifically responded to p225–256, p233–264 and p241–272 Cry j 2‐overlapping peptides (Fig. 3d). Therefore, B6pp30 was thought to respond to the common core epitope contained in p241–256 (GRENSRAEVSYVHVNG) (Table 1). On the other hand, B6pp45 specifically responded to p353–384 and p357–388 Cry j 2‐overlapping peptides (Fig. 3e). Therefore, B6pp45 was thought to respond to the common core epitope contained in p357–384 (LTSGKIASCLNDNANGYFSGHVIPACKN) (Table 1).

**Table 1:** The epitope peptide sequences corresponding to T‐cell lines

(a) SCR1	p193–225 of Cry j 1	FNHHKVMLLGHDDAYS**DDKSMKVTVAFNQFGPN**
	p201–232 of Cry j 1	LGHDDAYS**DDKSMKVTVAFNQFGPN**CGQRMPR
	p209–240 of Cry j 1	**DDKSMKVTVAFNQFGPN**CGQRMPRARYGLVHV
(b) BaCR1	p257–288 of Cry j 1	SNPTILSEGNSFTAPN**ESYKKQVTIRIGCKTS**
	p265–296 of Cry j 1	GNSFTAPN**ESYKKQVTIRIGCKTS**SSCSNWVW
	p273–304 of Cry j 1	**ESYKKQVTIRIGCKTS**SSCSNWVWQSTQDVFY
(c) BaCR2	p225–256 of Cry j 2	EDLICGPGHGISIGSL**GRENSRAEVSYVHVNG**
	p233–264 of Cry j 2	HGISIGSL**GRENSRAEVSYVHVNG**AKFIDTQN
	p241–272 of Cry j 2	**GRENSRAEVSYVHVNG**AKFIDTQNGLRIKTWQ
(d) B6pp30	p225–256 of Cry j 2	EDLICGPGHGISIGSL**GRENSRAEVSYVHVNG**
	p233–264 of Cry j 2	HGISIGSL**GRENSRAEVSYVHVNG**AKFIDTQN
	p241–272 of Cry j 2	**GRENSRAEVSYVHVNG**AKFIDTQNGLRIKTWQ
(e) B6pp45	p353–384 of Cry j 2	ISLK**LTSGKIASCLNDNANGYFSGHVIPACKN**
	p357–388 of Cry j 2	**LTSGKIASCLNDNANGYFSGHVIPACKN**LSPS

The bold font means the core peptide sequences common to three epitopes.

### T‐cell lines showed proliferative responses to TG‐rice seed extract

**Figure 4:**
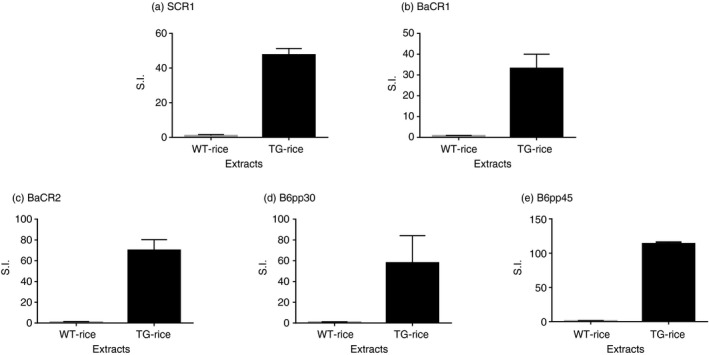
The proliferative responses of T‐cell lines to non‐transgenic wild‐type rice (WT‐) or transgenic rice (TG‐rice) seed extract. The proliferative responses of five types of T‐cell lines (a–e) to WT‐ or TG‐rice seed extract were determined by ^3^H‐thymidine incorporation assay, as described in the legend of Fig. 3.

**Figure 5:**
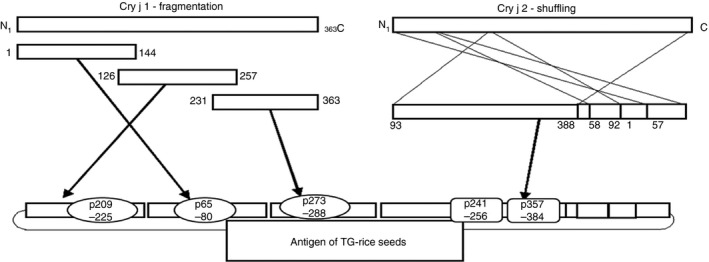
Antigen mapping in transgenic rice (TG‐rice) seeds. The antigen mapping shows the locations of epitope peptides that were proved to be expressed in TG‐rice seeds.

The proliferative response of each T‐cell line to TG‐rice seed extract was examined (Fig. 4). Every T‐cell line showed a positive proliferative response to TG‐rice seed extract compared with the response to WT‐rice seed extract, demonstrating that TG‐rice seeds certainly expressed T‐cell epitopes that the five types of T‐cell lines could respond to. TG‐rice seeds were shown to express p209–225 and p273–288 epitopes of Cry j 1 with antigenicity, based on the proliferative responses of T‐cell lines SCR1 and BaCR1 (Fig. 4; Table 1). The TG‐rice seeds were genetically engineered to contain the whole amino acid sequences of the Cry j 1 and Cry j 2 antigens in the endosperm: the Cry j 1 gene was divided into three overlapping fragments (Fragment 1: p1–144; Fragment 2: p126–257; and Fragment 3: p231–335), and the Cry j 2 gene was shuffled. The certain existence of p209–225 and p273–288 epitopes of Cry j 1 in the distinct fragments suggested that TG‐rice seeds contained Fragment 2: p126–257 and Fragment 3: p231–335 (Fig. 5). Although we did not establish a T‐cell line from C3H/He mice, major T‐cell epitopes to which T‐cells in Cry j 1‐immunized C3H/He mice reacted were assumed to be amino acid sequences common to p49–80, p57–88 and p65–96 overlapping peptide‐antigens of Cry j 1 (Fig. 2c). The proliferative response of T‐cells in Cry j 1‐immunized C3H/He mice to TG‐rice seed extract (Fig. 1c) suggested that TG‐rice seeds expressed the common core epitope of p65–80 (DRPLWIIFSGNMNIKL). P65–80 was located in Fragment 1: p1–144 (Fig. 5). Therefore, the expression of all three fragments in TG‐rice seeds indicated that they contained the whole Cry j 1 amino acid sequence. Similarly, based on the proliferative responses of T‐cell lines BaCR2, B6pp30 and B6pp45 to TG‐rice seed extract, it was proven that TG‐rice seeds expressed p241–256 and p357–384 epitopes of Cry j 2 with antigenicity (Fig. 4; Table 1). Based on the structural precondition that Cry j 2 amino acid sequences are shuffled but not intermittent in TG‐rice seeds, the certain expression of two T‐cell epitopes of Cry j 2 in TG‐rice seeds indicated that TG‐rice seeds contained the whole Cry j 2 amino acid sequence (Fig. 5). Therefore, we postulated that TG‐rice seeds expressed the whole Cry j 1 and Cry j 2 amino acid sequences.

### TG‐rice seeds retain antigenicity to T‐cells even after boiling at 100° for 30 min

**Figure 6:**
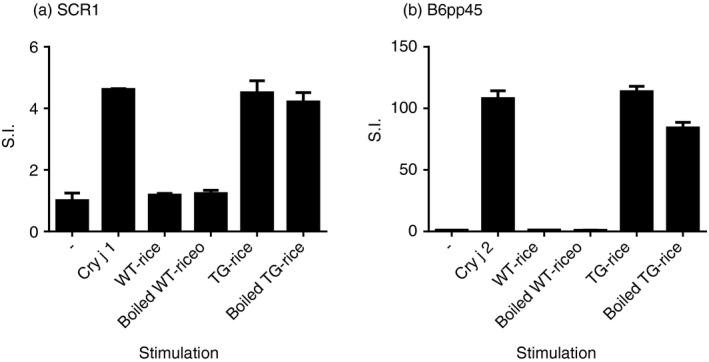
Transgenic rice (TG‐rice) seeds retain antigenicity even after boiling. The proliferative responses of Cry j 1‐specific T‐cell line SCR1 (a) and Cry j 2‐specific T‐cell line B6pp45 (b) to boiled non‐transgenic wild‐type rice (WT‐) or TG‐rice seed extract were determined by ^3^H‐thymidine incorporation assay as described in the legend of Fig. 3.

Cry j 1‐specific T‐cell line SCR1 and Cry j 2‐specific T‐cell line B6pp45 proliferated following treatment with boiled‐TG‐rice seed extract (Fig. 6). These results indicated that TG‐rice seeds retained antigenicity to T‐cells, even after boiling.

## Discussion

Our study has demonstrated that TG‐rice seed extract activated all Cry j 1‐ and Cry j 2‐specific T‐cells in four mouse strains immunized with Cry j 1 or Cry j 2. We further identified various types of T‐cells induced in Cry j 1‐ or Cry j 2‐immunized mice by using overlapping peptides spanning the Cry j 1 or Cry j 2 sequences. Detection of a positive proliferative response of the spleen cells, including various types of T‐cells to TG‐rice seed extract, suggested that TG‐rice seeds contained various epitope peptides of Cry j 1 and Cry j 2. However, these results did not necessarily confirm that TG‐rice seeds contained a specific epitope of Cry j 1 or Cry j 2. For example, the number of major T‐cell epitope sites was two in the Cry j 1‐immunized B10.S mice. However, a positive proliferative response of the spleen cells in the Cry j 1‐immunized B10.S mice to TG‐rice seed extract does not guarantee that TG‐rice seeds contain both epitopes. Even if only one epitope was expressed in the TG‐rice seed, a positive proliferative response of the spleen cells would occur. Therefore, T‐cell lines that selectively react with specific epitopes were required. Previously, T‐cell lines of Cry j 1, Cry j 2 and Japanese cypress pollen allergens Cha o 1 and Cha o 2 have been reported.^12^, ^13^, ^14^, ^15^, ^16^, ^17^ To specify T‐cell epitopes expressed in TG‐rice seeds, we established T‐cell lines and examined their proliferative responses to TG‐rice seed extract. The evident proliferative responses of T‐cell lines to TG‐ rather than WT‐rice seed extract definitively demonstrated that TG‐rice seeds contained specific T‐cell epitopes of Cry j 1 and Cry j 2. TG‐rice seeds were shown to express at least four T‐cell epitopes. Considering that the Cry j 1 gene was divided into three overlapping fragments and the Cry j 2 amino acid sequences were shuffled but not intermittent in TG‐rice seeds, the proliferative response of T‐cells in Cry j 1‐immunized C3H/He mice and the established T‐cell lines suggested that TG‐rice seeds expressed the whole Cry j 1 and Cry j 2 amino acid sequences. Therefore, our study indicated the possibility that the antigens expressed in TG‐rice seeds could target all types of Cry j 1‐ or Cry j 2‐specific T‐cells. Furthermore, it was proven that TG‐rice seeds retained antigenicity, even after boiling at 100° for 30 min. It was important to establish whether the polypeptides in TG‐rice seeds were stable after boiling, as rice seeds are usually eaten in the form of steamed rice. We believe that TG‐rice seeds will be an effective immunotherapeutic vehicle in T‐cell epitope‐based peptide immunotherapy.^18^

In Japan, SCIT and SLIT have been applied to treat JC pollinosis. However, because a crude or standardized vaccine is adopted in both treatments, the high doses of allergens has been associated with adverse events, including anaphylactic reactions. In a previous study, we used the basophil activation test to prove that recombinant allergens in TG‐rice seeds would be much safer than the antigens included in SCIT and SLIT against JC pollinosis and would decrease the risk of IgE‐mediated adverse events.^8^ Thus, it appears advantageous to administer oral immunotherapy with TG‐rice seeds.

The efficacy of a seed‐based peptide vaccine taken orally in humans has not been verified. Antigens are generally degraded in the gastrointestinal tract before arrival at the mucosal immune cells in gut‐associated lymphoid tissue due to exposure to the harsh environment in the stomach (low pH and pepsin).^19^ However, the recombinant proteins in protein bodies in the endosperm showed greater resistance to pepsin.^6^ When orally delivered via cereal seeds such as rice grains, bio‐encapsulation of the antigens within the double barriers of protein bodies and the cell walls characteristic of plant cells prove advantageous in that they protect the antigen from proteolysis.^19^ Thus, rice grains are advantageous in terms of enabling efficient delivery to the gut‐associated lymphoid tissue. Mice fed TG‐rice seeds daily for 3 weeks and then challenged with crude JC pollen allergen showed marked suppression of allergen‐specific CD4^+^ T‐cell proliferation, IgE and IgG levels compared with mice fed non‐transgenic rice seeds.^5^ The sneezing frequency and infiltration of inflammatory cells, such as eosinophils and neutrophils, were also significantly reduced in the nasal tissue,^5^ suggesting that oral administration of TG‐rice seeds induces immune tolerance against JC pollinosis. However, the mechanism of oral immune tolerance might be different from SCIT or SLIT. It was reported that oral immunotherapy was more efficacious than SLIT for the treatment of milk and peanut allergies.^20^, ^21^, ^22^ Milk, egg and peanut oral immunotherapy have consistently shown successful desensitization, although longer‐lasting tolerance does not appear likely at this stage of investigation.^23^ The most effective administration route of allergens expressed in TG‐rice seeds for the treatment of JC pollinosis should be investigated. Nevertheless, we consider that a rice‐based allergy vaccine taken orally is the best route if successful mucosal immune tolerance can be obtained. Oral immunotherapy with TG‐rice seeds may contribute to health economics as it would require neither food processing nor allergen component extraction from the rice seeds. Furthermore, oral immunotherapy with TG‐rice seeds might improve treatment adherence because rice is a principal food of the Japanese. In the near future, we will investigate the safety and the effectiveness of TG‐rice seeds as seed‐based oral vaccines in a double‐blind, placebo‐controlled study of patients with JC pollinosis.

## Disclosure

The authors have no conflicts of interest to declare.

## Author contributions

SS designed the study. ST and SS conducted the experiments. ST, TE, DA and SS interpreted the results. ST wrote the paper. YW, HT, KO and FT contributed to the extraction from the transgenic rice seeds. NO and HK supervised the study. All authors have read and approved the final manuscript.

The authors are grateful to Mayumi Tsuda and Yoko Natake for their technical assistance, and to Akiko Itasaka for her clerical work related to this study.
